# The Relationship Between Fibrinogen and Glycated Hemoglobin (HbA1c) in Diabetic Foot Ulcers

**DOI:** 10.7759/cureus.67174

**Published:** 2024-08-19

**Authors:** Kiran Notu, Jayakumar KT, Raji Rajesh Lenin, Nikhil Kumar G, JS Kumar

**Affiliations:** 1 General Medicine, Sri Ramaswamy Memorial (SRM) Medical College Hospital and Research Centre, Kattankulathur, IND; 2 Medical Research, Sri Ramaswamy Memorial (SRM) Medical College Hospital and Research Centre, Kattankulathur, IND

**Keywords:** ankle-brachial index, wagner classification, hba1c, fibrinogen, diabetic foot ulcer

## Abstract

Introduction: Diabetic foot ulcers (DFUs) are a common complication of diabetes that affects patients' quality and prognosis of life. The study aims to assess the correlation between fibrinogen and glycated hemoglobin (HbA1c) in DFUs at the first and sixth months and to compare fibrinogen levels with Wagner classification in DFU patients.

Methods: This observational study was conducted at SRM Medical College Hospital and Research Centre from January 2021 to July 2022. Fifty diabetes patients with DFUs were selected, and informed consent was obtained before the study started. Blood samples were collected from all the participants for HbA1C, serum fibrinogen, hemoglobin, and white blood cells. In this study, data were entered into MS Excel (Microsoft Corporation, Redmond, WA) and analyzed using SPSS version 24 (IBM Corp., Armonk, NY). ANOVA and Pearson's correlation were used to examine the relationships between serum fibrinogen levels and clinical parameters.

Results: Among 50 patients, the females were 16 (32%), and the males were 34 (68%). Most patients (34%) were in the 56-60 age group. Twenty patients had diabetes for 10 years, and 24 were diabetic for 11-15 years. The ankle-brachial index (ABI score) was mild in 14 patients (28%), moderate in 28 patients (56%), and normal in eight patients (16%). There is a significant difference in comparison between the Wagner classification and ABI. A significant difference was observed in fibrinogen at the first and sixth months between HbA1c first, third, and sixth months. Significant differences were also observed in fibrinogen and ABI in the first and sixth months.

Conclusion: Key findings include significant differences between fibrinogen and HbA1c levels (p < 0.0001) and a strong association between fibrinogen levels and ABI scores (p < 0.0001), underscoring fibrinogen's potential as an early marker for glycemic control and peripheral arterial disease in DFU patients. We concluded that simple fibrinogen estimation helps predict glycemic control in diabetic patients with DFUs.

## Introduction

Over the past 50 years, substantial lifestyle and globalization changes have profoundly influenced politics, the environment, society, and human behavior. Among the most significant impacts is the sharp rise in obesity and diabetes prevalence. Once primarily a concern in developed nations, diabetes has become a widespread issue affecting both developed and developing countries [1]. The increasing prevalence of diabetes mellitus poses a major public health challenge, straining patients, caregivers, healthcare systems, and society at large. As of 2017, there were 425 million cases of diabetes globally, with projections estimating that this number will rise to 629 million by 2045 [2,3].

Diabetes is a chronic condition marked by elevated blood glucose levels and protein and fat metabolism disruptions. This occurs due to insufficient insulin production by the pancreas, ineffective utilization of available insulin, or both, leading to elevated blood sugar levels [4,5]. Type 1 diabetes mellitus and type 2 diabetes mellitus (T2DM) are distinct disorders with varying clinical presentations and disease trajectories. In children, type 1 diabetes is often characterized by polyuria, polydipsia, and diabetic ketoacidosis (DKA), which occurs in about one-third of cases. In adults, type 1 diabetes mellitus may present differently, and DKA can also occur in T2DM, particularly in certain ethnic and racial groups. The classification of diabetes types can be unclear at initial presentation [6].

Diabetes can lead to symptoms such as polyuria, polydipsia, polyphagia, severe hyperglycemia, and weight loss. Undiagnosed diabetes may result in unexplained weight loss, fatigue, irritability, and discomfort, with some mild symptoms potentially going unnoticed [7]. Beyond these symptoms, diabetes mellitus is associated with numerous complications, including coronary heart disease, stroke, peripheral arterial disease, diabetic kidney disease, retinopathy, and peripheral neuropathy. It is also linked to various age-related liver disorders and infections, further complicating the health issues faced by diabetic patients [8]. Particularly severe complications in the lower limbs include diabetic foot ulcers (DFUs), which arise from poor foot care, peripheral vascular disease, and inadequate glycemic control [9,10].

Common laboratory tests for evaluating ulcers include fasting blood sugar, glycated hemoglobin (HbA1c) levels, complete metabolic panel, complete blood count, erythrocyte sedimentation rate, and C-reactive protein. Elevated plasma fibrinogen levels have been observed in diabetic patients, with those suffering from DFUs exhibiting higher fibrinogen levels compared to those without ulcers. Additionally, diabetic patients tend to have shorter fibrinogen half-lives and faster clearance rates [11]. Early detection and optimal treatment of DFUs improve prognosis; however, delays in treatment can lead to severe outcomes, including amputation. Patients with persistent diabetic ulcers face a high risk of rehospitalization and prolonged hospital stays [12].

Despite fibrinogen being an established marker of vascular inflammation and endothelial dysfunction in diabetes, there is limited evidence of the relationship between fibrinogen levels and HbA1c in the context of DFUs.

Despite fibrinogen's known role as a marker of vascular inflammation in diabetes, its relationship with HbA1c in DFUs remains underexplored. Literature indicates that elevated fibrinogen levels are common in diabetes and that individuals with DFUs tend to have higher fibrinogen levels than those without ulcers [13,14]. Elevated fibrinogen levels and altered fibrinogen metabolism may play a role in foot ulcer development and progression. This study seeks to elucidate the connection between fibrinogen and HbA1c levels and determine whether fibrinogen could be an early indicator of glycemic control and ulcer severity, potentially guiding more effective interventions for DFUs.

This study investigates the correlation between serum fibrinogen levels and HbA1c at the initial visit and after six months to identify potential prognostic markers for diabetic complications. Additionally, it compares fibrinogen levels with the Wagner classification to assess their association with ulcer severity. Given the rising global prevalence of diabetes and its complications, understanding this relationship could enhance patient care and management strategies for DFUs. This study explores the association between serum fibrinogen levels and HbA1c in DFUs to enhance understanding and improve patient care standards. Specifically, the study investigates the correlation between fibrinogen and HbA1c at the initial visit and after six months, seeking to identify potential prognostic markers for diabetic complications. Additionally, we compare fibrinogen levels with the Wagner classification in DFU patients.

## Materials and methods

This observational study was conducted at SRM Medical College Hospital and Research Centre from January 2021 to July 2022. Fifty patients with diabetes mellitus and DFUs were selected for the study. The study was conducted in accordance with ethical standards, and approval was granted by SRM Medical College Hospital and Research Centre (ethical clearance number: 2161/IEC/2021). Informed consent was obtained from each participant before the study commenced, ensuring they were fully aware of the study's purpose, procedures, and potential risks or benefits.

Inclusion criteria

We included known T2DM patients with DFUs, male and female patients of age >45, and DFU patients with HbA1C ≥8.

Exclusion criteria

T2DM patients without DFUs and patients on antiplatelets and antithrombotics were excluded.

Sample collection

Blood samples were collected from all the participants for HbA1C, serum fibrinogen, hemoglobin, and white blood cells. The ankle-brachial index (ABI) was performed by measuring the systolic blood pressure from both brachial arteries and the dorsalis pedis and posterior tibial arteries after the patient had rested in the supine position for 10 minutes. The systolic pressures are recorded with a handheld 5- or 10-MHz Doppler instrument.

Sample grading

Grading of DFU according to Wagner's classification is as follows: grade 0 for intact skin in patients who are at risk, grade I for superficial ulcers with exposed subcutaneous tissue, grade II for exposed tendon and deep structures, grade III ulcers extend to the deep tissue and have associated soft tissue abscesses or osteomyelitis, grade IV for ulcers includes feet with partial gangrene, and grade V for feet ulcers with more excessive gangrenous tissue.

Wagner classification

Correlation between serum fibrinogen with HbA1C level, age, sex, ABI, and duration of diabetes mellitus in patients with DFU at the time of first visit and follow-up after six months was done. HbA1C was done at the first visit in the third and sixth months. Fibrinogen levels among Wagner classification in DFUs were compared.

Wagner's categorization was used to categorize DFU. The brachial arteries, dorsalis pedis, and posterior tibial arteries were used to calculate the ABI. At the initial visit in the third and sixth months, HbA1C was estimated.

In patients with DFUs, the relationship between serum fibrinogen and HbA1C level, age, sex, ABI, and duration of diabetes mellitus was evaluated at the initial visit and at the follow-up appointment after six months. Also, fibrinogen levels in DFU patients were compared with the Wagner grading.

Data analysis

The data were entered into MS Excel and calculated. All demographic data were presented as frequency and percentage. ANOVA and Pearson's correlation were used to analyze the data.

## Results

Key results from 50 patients, including 32% females and 68% males, revealed a predominant age group of 56-60 years (34%). The duration of diabetes varied, with 40% having diabetes for 11-15 years. According to the Wagner classification, 44% had grade 3 DFU, 36% had grade 4, and 20% had grade 5. ABI scores indicated mild impairment in 28%, moderate impairment in 56%, and normal readings in 16%. Significant differences were found between the Wagner classification and ABI scores (p < 0.0001), with a 95% confidence interval (CI) for the mean difference suggesting a strong association with peripheral arterial disease. Fibrinogen levels in the first month showed significant differences from HbA1c levels in the first, third, and sixth months (all p values <0.0001), with effect sizes (Cohen's d) indicating large practical significance (Tables 1-3).

**Table 1:** Demographic data of the study ABI: ankle-brachial index

Category		Frequency	Percentage
Gender	Female	16	32.0%
	Male	34	68.0%
Age group	<50	5	10.0%
	51-55	11	22.0%
	56-60	17	34.0%
	61-65	8	16.0%
	>66	9	18.0%
Diabetic duration	<10	20	40.0%
	11-15	24	48.0%
	>16	6	12.0%
Wagner classification (1-5)	Grade 3	22	44.0%
	Grade 4	18	36.0%
	Grade 5	10	20.0%
ABI	Mild	14	28.0%
	Moderate	28	56.0%
	Normal	8	16.0%

**Table 2:** Comparison of Wagner classification with ABI ABI: ankle-brachial index

Wagner classification (1-5)	ABI			p value
	Mild	Moderate	Normal	
Grade 3	14 (63.6%)	0	8 (36.4%)	<0.0001
Grade 4	0	18 (100%)	0	
Grade 5	0	10 (100%)	0	

**Table 3:** Association of fibrinogen in the first month with HbA1c, ABI, diabetic duration, and age HbA1c: glycated hemoglobin; ABI: ankle-brachial index

Fibrinogen in the first month	Pearson’s correlation	p value
HbA1c: first month	0.659	<0.0001
HbA1c: third month	0.631	<0.0001
HbA1c: third month	0.577	<0.0001
ABI	-0.735	<0.0001
Diabetic duration	-0.008	0.955
Age	-0.136	0.345

**Figure 1:**
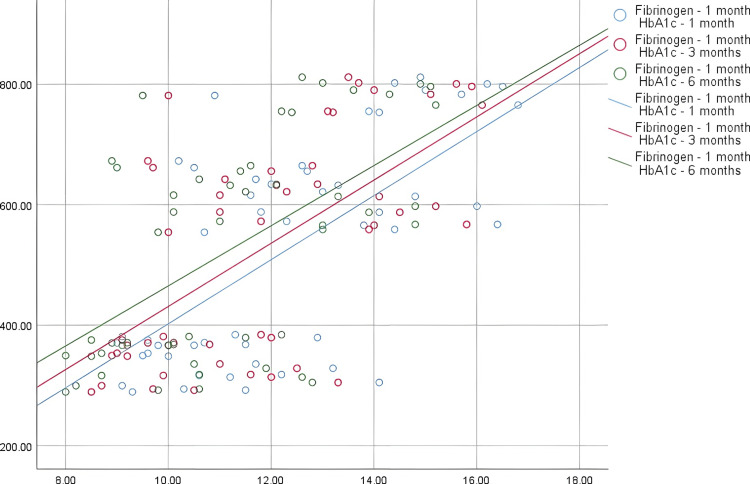
Association between fibrinogen in the first month and HbA1c levels in the first, third, and sixth months HbA1c: glycated hemoglobin

There is a significant difference in comparison between the Wagner classification and ABI (p = <0.0001) (Table 2). This indicates a strong association between the severity of DFUs and peripheral arterial disease. Regarding biomarkers, fibrinogen levels in the first month showed a statistically significant difference compared to HbA1c levels in the first, third, and sixth months, with all comparisons yielding p values of <0.0001 (Figure 1). This underscores the potential of fibrinogen as a reliable early indicator of glycemic control.

There is a notable difference between fibrinogen levels in the first month and ABI (p < 0.0001). There is no significant variation in fibrinogen levels in the first month between diabetic duration and age (Table 3). This suggests that fibrinogen levels are more closely related to peripheral arterial disease than to the duration of diabetes or age of the patients.

**Figure 2:**
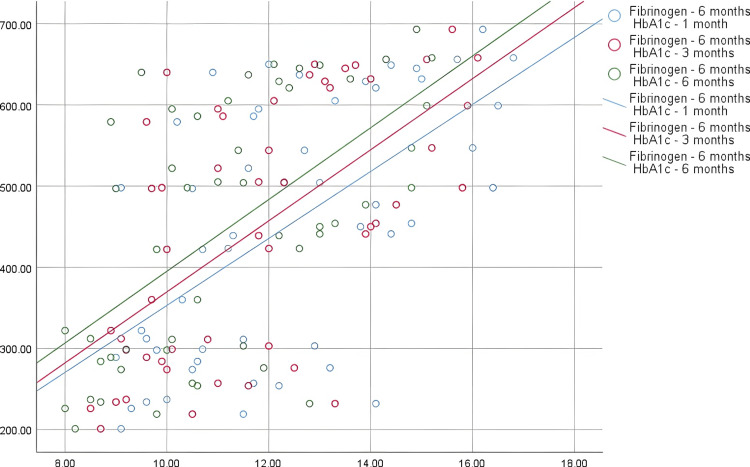
Association between fibrinogen in the sixth month and HbA1c levels in the first, third, and sixth months HbA1c: glycated hemoglobin

A marked distinction was observed in fibrinogen levels at the sixth month when compared to HbA1c levels at the first (p < 0.0001), third (p < 0.0001), and sixth (p < 0.0001) months (Figure 2). This highlights the strong correlation between long-term fibrinogen levels and glycemic control as measured by HbA1c.

Fibrinogen levels also differed significantly from ABI scores (p < 0.0001), with a 95% CI further supporting a strong association. At six months, fibrinogen levels remained significantly correlated with HbA1c (all p values <0.0001) and ABI (p <0.0001), with no significant variation relative to diabetes duration or age (p >0.05). This highlights the potential of fibrinogen as a robust marker for inflammation and peripheral arterial disease, more so than diabetes duration or patient age (Table 4). However, fibrinogen levels at this time point do not vary significantly with the duration of diabetes or age, suggesting that these factors may not influence fibrinogen levels as strongly as ABI.

**Table 4:** Association of fibrinogen in the sixth month with HbA1c, ABI, diabetic duration, and age HbA1c: glycated hemoglobin; ABI: ankle-brachial index

Fibrinogen in the sixth month	Pearson’s correlation	p value
HbA1c: first month	0.592	<0.0001
HbA1c: third month	0.609	<0.0001
HbA1c: sixth month	0.591	<0.0001
ABI	-0.704	<0.0001
Diabetic duration	0.028	0.845
Age	-0.088	0.543

## Discussion

Serum fibrinogen is an inflammatory marker that has a significant role in the etiopathogenesis of inflammation, atherosclerosis, thrombogenesis, and the progress of vascular complications in T2DM. Macroangiopathy is a form of accelerated atherosclerosis affecting the carotid, coronary, and peripheral arteries, and DFU is rising due to this occurrence. Patients with diabetes have higher plasma fibrinogen production levels [11].

A previous study found that DFUs were more common in males, with 57.5% of males affected compared to 42.4% of females. In our current study, 68% of the patients were male, and 32% were female [11]. Using Wagner's classification, the previous study reported that 39% of patients had grade 3 DFU, 49% had grade 4, and 9% had grade 5. In our study, 44% had grade 3 DFU, 36% had grade 4, and 20% had grade 5 DFU.

A significant correlation between fibrinogen serum levels and HbA1c (p = 0.001, r = 0.387) and between fibrinogen serum levels and ABIs (p = 0.008, r = 0.454) was found in the previous study [11]. Our study also observed a significant relationship (p < 0.0001) between fibrinogen levels at the first and sixth months and HbA1c levels at the study's first, third, and sixth months. Studies found that fibrinogen serum levels in DFU patients were considerably greater in those with poor glycemic control and were linked with HbA1c. Our study also noted a similar observation and an association between HbA1c and fibrinogen.

In a study conducted by Ali et al. [13], it was observed that the serum levels of omentin-1 were significantly different in individuals with DFUs compared to the control group. Similarly, fibrinogen levels were significantly different in the DFU group compared to the control group. The study also revealed a significant negative correlation between omentin-1 and HbA1c levels, while a significant positive correlation was observed between fibrinogen and HbA1c levels. A similar relationship was established in our study, too, where a significant and positive relationship between HbA1c and fibrinogen was noted (p < 0.0001).

In a study by Li et al. [14], 89 (59.3%) males and 61 (40.7%) females were reported, which is similar to the demographics of our study, where 68% were males and 32% were females. Grade 3 had 31 patients, grade 4 had 29, and grade 5 had 37 patients. In our study, we had 22 patients in grade 3, 18 in grade 4, and 10 in Wagner's grade 5. Their study concluded that fibrinogen is a good predictor of the severity and progression of DFU. This positive and strong association of fibrinogen was noted in our study, with a p value of 0.0001 and a significantly positive Pearson coefficient.

The study by Bembde reported that T2DM patients had significantly higher plasma fibrinogen levels (656 ± 130 mg/dL) compared to the control group (324 ± 139 mg/dL). Age and glycosylated hemoglobin (r = 0.49) significantly correlated with fibrinogen levels in people with diabetes, but there was no correlation with sex [15].

Unlike this study, we found no relationship between age and fibrinogen measured at the first-month and sixth-month follow-up. Bembde's studies concluded that hyperfibrinogenemia was more common in people with T2DM, and HbA1c values were independently correlated with fibrinogen levels [15]. Our study found a similar positive correlation between the two parameters, HbA1c and fibrinogen, where the poorer the glycemic control, the higher the fibrinogen levels.

Smith-Strøm et al. [16] conducted a study to assess the relationship between serum fibrinogen, serum albumin, and platelet values in 108 T2DM DFU patients. Fifty-four were T2DM without DFU, and 54 were with DFU. The serum fibrinogen mean in DFU was 412 ± 13.4, and in diabetic persons, it was 296.5 ± 13.6. The study concluded that a significant rise in serum fibrinogen in diabetic patients is associated with disease severity. The results follow our study, where elevated fibrinogen levels were noted with poor glycemic control.

Yang et al. [17] evaluated 482 outpatients with T2DM who received an ABI examination from 2010 to 2017. Among multiple parameters assessed, ABI and foot ulcer status were studied. The study deduced that low ABI patients had a significantly higher foot ulcer rate (p = 0.039). The present research echoed these results, and there was a significant difference in comparison between the Wagner classification and ABI (p < 0.0001).

The elevation of serum fibrinogen in DFUs is primarily due to chronic inflammation and endothelial dysfunction. These conditions stimulate the liver to produce more fibrinogen, aiding wound healing and tissue repair. Hyperglycemia, associated with diabetes, induces chronic inflammation and oxidative stress, further increasing fibrinogen production as part of the body's inflammatory response. While the provided data do not explicitly indicate whether fibrinogen levels decreased as DFUs settled, the significant differences observed between fibrinogen levels and HbA1c at various time points suggest potential changes over time that might correlate with the healing or progression of DFUs.

We acknowledge the potential limitations of the study, such as the small sample size and the risk of confounding variables, such as variations in patient adherence to treatment or comorbid conditions. To determine whether fibrinogen levels decreased as DFUs settled, future studies will be conducted to analyze the trends in fibrinogen levels throughout the entire study period and correlate these with the clinical outcomes of the DFUs. Future research should focus on larger, multicenter trials to validate these findings and explore additional factors that might influence the fibrinogen-HbA1c relationship, including genetic and lifestyle variables.

## Conclusions

The study showed a positive correlation between fibrinogen measured in the first and sixth months and HbA1c (measured in the first, third, and sixth months). This study's findings could enable early identification of high-risk DFU patients through fibrinogen levels, improving glycemic control and ulcer management. Incorporating fibrinogen assessments in clinical practice may enhance predictive accuracy and treatment outcomes. There was a significant negative correlation noted between fibrinogen and ABI. No significant association was noted between fibrinogen and age and duration of diabetes, but there was a significant association between Wagner's grading of DFU and branchial ankle index. Thus, this study provides valuable insights into the relationship between fibrinogen and HbA1c in DFUs, contributing to the understanding of inflammatory markers in diabetes management.
